# Comparison of a long-read amplicon sequencing approach to short-read amplicons for microbiome analysis

**DOI:** 10.1128/spectrum.02776-25

**Published:** 2026-05-19

**Authors:** Brandon O'Sullivan, Katherine W. Herbst, Alexander H. Hogan, Michele Maltz-Matyschsyk, Justin D. Radolf, David Lawrence, Michael A. Lynes, Juan C. Salazar, Joerg Graf, The Connecticut Children’s COVID Collaborative, Michael Brimacombe

**Affiliations:** 1 Pacific Biosciences Research Center, University of Hawaiʻi at Mānoa, Honolulu, Hawaii, USA; 2 Connecticut Children’s Research Institute, Hartford, Connecticut, USA; 3 Division of Hospital Medicine, Connecticut Children’s, Hartford, Connecticut, USA; 4 Department of Pediatrics, University of Connecticut School of Medicine 12227, Farmington, Connecticut, USA; 5 Department of Molecular and Cell Biology, University of Connecticut 124501 , Storrs, Connecticut, USA; 6 Department of Immunology, University of Connecticut School of Medicine 12227, Farmington, Connecticut, USA; 7 Department of Genetics and Genome Sciences, University of Connecticut School of Medicine 12227, Farmington, Connecticut, USA; 8 Department of Medicine, University of Connecticut School of Medicine 12227, Farmington, Connecticut, USA; 9 Department of Molecular Biology and Biophysics, University of Connecticut School of Medicine 12227, Farmington, Connecticut, USA; 10 Department of Biomedical Sciences, University at Albany 1084, Albany, New York, USA; 11 Division of Infectious Disease & Immunology, Connecticut Children’s, Hartford, Connecticut, USA; 12 Department of Pediatrics, Vanderbilt University Medical Center, Nashville, Tennessee, USA; Brigham Young University, Provo, Utah, USA

**Keywords:** microbiome, 16S RNA, rRNA, oral microbiome, human microbiome, DNA sequencing, amplicon sequencing

## Abstract

Most microbiome studies to date rely on sequencing short amplicons of the 16S rRNA gene on Illumina’s platforms. Because of the short read length, sequences often can be identified reliably only to the family or genus levels. Long-read sequencing with whole-length 16S rRNA sequencing can improve taxonomic resolution but often only to the species level. StrainID is an alternative approach that amplifies a large segment of the ribosomal operon, including the entire 16S rRNA gene, internal transcribed spacer, and a portion of the 23S rRNA gene. This longer amplicon is designed to allow ribotype-level classification. Although studies have demonstrated the utility of StrainID for several sample types, a direct comparison of StrainID to alternative approaches has not been done for saliva. Here, we compared the performance of StrainID to short-read amplicons with saliva samples as well as a synthetic mock DNA community. Short reads were amplified with primer pairs targeting the V1-V3 region of the 16S rRNA gene and were classified with several different taxonomic databases. We found that StrainID outperformed short reads not only in identifying amplicon sequence variants to the species level but also in demonstrating a key benefit with phylogenetic-based beta-diversity tests. Our results further build on establishing StrainID as a powerful method and specifically for its use with saliva samples.

**IMPORTANCE:**

The interpretation of microbiome composition studies is highly dependent on the methodologies chosen during experimental design, which affects factors such as resolution, throughput, cost, and accuracy. StrainID is an approach that can improve resolution while maintaining high-throughput and similar costs to short-read sequencing. The salivary microbiome represents a diverse community of microbes with links to a variety of health conditions and disease states. Closely related strains of bacteria can have drastically different effects on their host. Establishing StrainID as a valid approach for studying the salivary microbiome opens avenues for research that improve upon alternative methods by increasing sensitivity and accuracy compared to traditional short-read approaches.

## INTRODUCTION

Next-generation sequencing has revolutionized microbiome research, enabling rapid, highly accurate characterization of hundreds of microbial communities simultaneously (1, 2). However, most existing research has relied on short-read amplicon (SRA) sequencing approaches, focusing on small hypervariable regions within the 16S ribosomal RNA (rRNA) gene and, consequently, limiting taxonomic resolution to genus-level or higher classifications (3). Careful selection of primers for a specific region of the 16S rRNA gene or other genes (such as *rpoB* [4] and *gyrB* [5, 6], among others) can improve resolution for certain sample types by focusing on regions that are most variable for the abundant taxa in that environment, although this does not overcome all limitations of this approach (7). Shotgun or non-targeted metagenomics offers a potential solution to this problem by sequencing random fragments of all DNAs present in a sample. This approach provides much more data on the community, but it is more expensive and computationally taxing than short-read amplicon sequencing and generally requires both higher quality and quantities of DNA (8, 9). In addition, samples with a high proportion of host DNA, such as saliva, require effective host DNA depletion to make metagenomic sequencing economically feasible (10). One potential outcome of this approach is improved insights into the metabolic potential of the abundant members of the microbiome at the expense of reduced information on less abundant microorganisms (11). These low-abundance organisms can play important roles in determining the composition and function of microbiomes (8). Long-read sequencing technologies—such as those done with PacBio or Oxford Nanopore instruments—have the potential to strike a middle ground by amplifying and sequencing the entire 16S rRNA gene. This typically increases resolution to species-level classification, representing a major improvement over SRA sequencing, but fails to identify strains as often as metagenomics can (3). Strains from the same species can differ in metabolic function (12), toxin production (13), and antibiotic resistance (14), each with their own potential effects on the host. As such, choosing a technology that allows one to analyze a large enough cohort to detect compositional differences between microbiomes and with sufficient resolution at the sequence level to reveal potential differences is key for the success of clinical studies.

**Fig 1:**
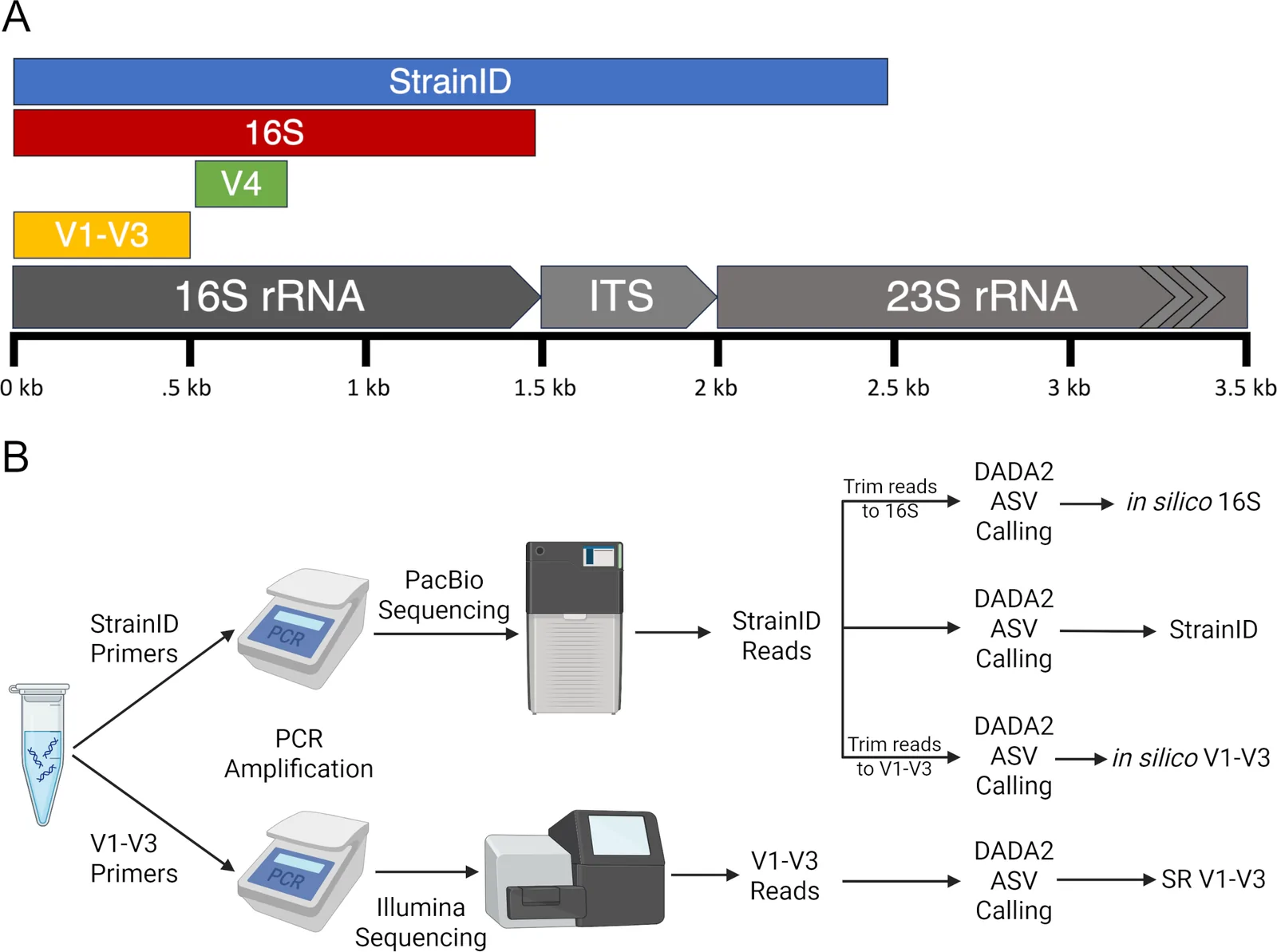
Experimental design. (**A**) Size and position of the amplicons used in this study relative to a standard ribosomal RNA operon. (**B**) Sample processing pipeline for downstream data analysis. Every sample was processed with all methods to generate four total data sets. “Figure 1: Experimental design” created in BioRender. Graf, J. (https://BioRender.com/6rqw04p) is licensed under CC BY 4.0.

StrainID, an approach commercialized by Intus Biosciences (formerly Shoreline Biome), attempts to improve on whole-length 16S rRNA sequencing at a similar cost by including more of the ribosomal RNA operon. Compared to the approximately 1,500 bp length of the entire 16S rRNA gene, StrainID uses custom primers to produce an amplicon nearly 2,500 bp in length that includes the internal transcribed spacer (ITS) region and a portion of the 23S rRNA gene (Fig. 1A) (15, 16). Most standard microbiome databases are not designed for sequences spanning the ribosomal operon. Athena is a proprietary custom database designed specifically for use with StrainID and consists of high-quality rRNA operon sequences retrieved from published genomes. Other publicly available databases such as MIrROR (17) and GROND (18) are also designed for 16S-ITS-23S rRNA amplicons by deriving operon sequences from published genomes. With Athena, StrainID is reported to provide strain-level classifications (15). Because strains can be defined by differences in other key genes without any changes in the rRNA operon sequence, we term these ribotypes instead (19). Herein, we refer to these ribotypes as strains for consistency with their identification by classifiers. Nonetheless, ribotype-level classification represents a substantial improvement over species-level classification.

Most bacteria carry multiple copies of the ribosomal operon in their genome, and these copies frequently have different sequences, especially in the ITS region, often encoding transfer RNA (20). Past studies have shown that the StrainID approach can be used to identify distinct patterns of these sequences that co-occur, generating a “fingerprint” that can be used to identify and track specific bacteria that share the same set of rRNA sequences, or ribotypes, across samples (15, 16). To date, StrainID has been validated for several sample types, including fecal, human milk, and aquaculture water and swabs (15, 16, 21, 22).

The human oral microbiome, the second largest and second most diverse bacterial community in the human body, contains numerous medically significant bacteria (23, 24). The oral microbiome is temporally stable and can be influenced by environmental factors (25). Additionally, due to the prevalence of biofilms, the oral microbiome tends to be less susceptible than the gut microbiome to disruption by antibiotics (26). Changes in the relative abundances of certain bacterial taxa in saliva have been correlated with various systemic pathologies, including cardiovascular disease, rheumatoid arthritis, and Alzheimer’s disease (24). These findings, combined with the less invasive nature of saliva collection, raise the possibility that saliva could replace blood for diagnostic tests in some contexts (27, 28). However, approaches to identify these signals must be sensitive and specific. With short-read approaches, the V1–V3 hypervariable regions represent the section of the 16S rRNA gene that provides the greatest ability to resolve taxa associated with saliva, especially streptococci (29, 30). For Illumina sequencing, the length of this amplicon requires the use of Illumina’s 600-cycle kit, which generates paired-end 300 bp reads. The chemistry used in Illumina sequencing is associated with a decrease in read quality as the length increases, especially in the reverse read (31). On the original MiSeq, this problem is far more pronounced in the 600-cycle kit compared to even the 500-cycle kit, though this is less of a problem with newer Illumina systems (32). The decrease in quality can result in paired-end reads failing to join due to insufficient overlap, and reads with low quality may have to be discarded for failing quality filtering, especially when using denoised amplicons. In addition, longer reads that contain more variable sites than short reads can potentially provide greater sensitivity in distinguishing different cohorts, especially when using phylogenetic distance-based metrics (33).

Herein, we sought to compare the performance of StrainID with human saliva samples to V1–V3 amplicon sequencing. To increase confidence in our findings and their applicability, we confirmed the performance of StrainID with a mock DNA community representing diverse bacteria, including gram-positive and gram-negative organisms. Mock communities have been established as a way to verify the success of sequencing runs and estimate biases (34).

The V1–V3 regions are often used to study the salivary microbiome (29). As such, we used saliva samples to compare StrainID specifically against V1–V3 amplicons (Fig. 1B). All sets of amplicons were benchmarked with the mock community. In addition, we assessed the impact of using different databases for taxonomic assignment as there has been limited work assessing the importance of the reference databases for StrainID (15, 21). Finally, we sought to compare the resolving power of StrainID and V1–V3 with human saliva samples. For this comparison, we selected the collection method, which is a binary variable (swab and passive drool) with approximately equal representation of both groups in our data set.

## MATERIALS AND METHODS

### Biospecimen collection

Saliva was collected from 46 subjects, from birth to ≤21 years of age, enrolled in a prospective study at a single center between March 2021 and August 2022 (IRB approval #21-004). The study’s primary aim was to identify a biosignature of diagnostic value for multisystem inflammatory syndrome in children (MIS-C) (35). All patients were enrolled in Connecticut and had variable health statuses, including individuals with inflammatory conditions (viral respiratory infections, MIS-C, and Kawasaki disease) as well as non-inflammatory controls. Saliva was collected at the time of enrollment using the SalivaBio Infant’s Swab Collection kit for subjects from birth to <6 years of age (5001.8, 5001.5; Salimetrics, Pennsylvania, USA) or the SalivaBio Passive Drool Collection Aid (5016.04, Salimetrics) for subjects ≥6 years of age. In total, 21 samples were collected by passive drool, and 25 samples were collected by swab.

### Initial processing of saliva

Saliva samples were frozen without stabilizer and stored at −80°C for a minimum of 24 h and then thawed at room temperature for approximately 20 min. Debris was pelleted by centrifugation for 15 min at 200 rcf and 4°C. A 250 μL aliquot of the supernatant was held at 56°C for 30 min to heat-inactivate any present SARS-CoV-2. Samples were treated with 6.25 μL of Proteinase K, which was subsequently inactivated by heating samples to 95°C for 5 min. Processed saliva samples were stored at −80°C prior to DNA extraction.

### DNA extraction

DNA was extracted using the Shoreline Complete Lyse & Purify kit (Intus Biosciences, Farmington, CT, USA), which uses an alkaline lysis and magnetic bead purification. Extractions were performed according to the manufacturer’s protocol. Saliva samples were thawed on ice, and between 50 and 200 µL of saliva was pelleted by centrifugation at 5,500 rcf for 10 min. Pellets were resuspended in 50 μL nuclease-free water. To extract, samples were added to wells containing dried Lysis-1 and mixed with 50 μL of 0.4 M KOH. Samples were then heated to 95°C for 5 min to lyse cells. Next, 100 μL of purification buffer containing magnetic beads was added to each well. DNA was bound to the beads with a 50°C incubation step. Beads were bound to a magnetic rack, and the supernatant was removed. Following an ethanol rinse, DNA was suspended in a total of 40 μL of 1× TE buffer. To confirm successful extraction of bacterial DNA, samples were screened by qPCR targeting the 16S rRNA gene.

### PCR amplification and library prep

Extracted DNA was amplified with the Intus Biosciences Shoreline Complete StrainID Set A Amplify and Shoreline Wave V1–V3 PCR Amplify Set A kits for StrainID and V1–V3, respectively. Researchers interested in acquiring the kits can contact Mark Driscoll (mark.driscoll@intusbio.com) of Intus Biosciences to discuss potential collaboration. Amplifications were performed according to the manufacturer’s protocol and as previously described (16). Both kits have identical protocols, excluding thermocycler parameters. Ten microliters of DNA and 10 µL of PCR mix (Intus Biosciences) were added to each well containing dried barcoded primers (Table 1). As a control and to test for primer bias, we also amplified a ZymoBiomics Mock DNA community (catalog number D6305; Zymo Research, Irvine, CA, USA) with both amplicon types. This mock community contained equal concentrations of genomic DNA from eight diverse species of bacteria (*Listeria monocytogenes*, *Pseudomonas aeruginosa*, *Bacillus subtilis*, *Escherichia coli*, *Salmonella enterica*, *Lactobacillus fermentum*, *Enterococcus faecalis*, and *Staphylococcus aureus*) representing a wide range of GC content to help identify bias.

**TABLE 1:** Primer sequences

Primer name	Sequence (5′→3′)	Amplicons used in
27F	AGRRTTYGATYHTDGYTYAG	StrainID, V1–V3, 16S (*in silico* only)
515R	TYACCGCRRCKGCTGGCAC	V1–V3
StrainID_R	AGTACYRHRARGGAANGR	StrainID
1492R	TASVGHTACCTTGTTACCGACTT	16S (*in silico* only)

Amplicons were screened for successful amplification on a QIAxcel Advanced (Qiagen, Germantown, MD, USA), a capillary electrophoresis system, using the Fast Analysis protocol. For an approximate standardization, samples were pooled qualitatively based on the relative band intensity from the visualization of the QIAxcel Advanced to a standard and to other samples. Five microliters from each sample was combined into one of two pools: samples with a relatively intense band were added to the first pool (“dark”), and samples with faint bands were combined into the second pool (“light”). Both the light and dark pools were cleaned using the GeneRead Size Selection Kit (Qiagen) according to the manufacturer’s protocol and resuspended in 50 µL of elution buffer. The purity of the cleaned light and dark pools was verified by gel electrophoresis. Cleaned light and dark amplicons were quantified by Qubit (Thermo Fisher Scientific, Waltham, MA, USA). The light and dark pools were combined together by adding equal amounts of DNA per sample and were sent for sequencing. StrainID samples were sequenced on a PacBio Sequel IIe, while the V1–V3 samples were sequenced on an Illumina MiSeq with the v3 2 × 300 Kit.

### Data processing and statistical analysis

Raw sequences were first demultiplexed with Illumina BaseSpace for the V1–V3 amplicons and SBAnalyzer for the StrainID amplicons. To account for any biases from the primers, we generated *in silico* reads by trimming the StrainID reads based on the V1–V3 and full 16S primer sequences. Reads were error corrected with the software package DADA2 to generate exact sequences known as amplicon sequence variants (ASVs) (36). Spurious taxa were removed by filtering using a minimum relative abundance threshold based on the presence of unexpected taxa or chimeric sequences in the mock communities. This threshold was 0.225% for V1–V3 and 0.1% for all the other samples. Taxonomic assignment was performed with the Athena database and its built-in classifier with SBAnalyzer (16). Data analysis was performed in QIIME2 (2024.5) and R (37). Taxonomic assignment with additional databases was done in QIIME2 using pretrained Naïve Bayes classifiers for Silva 138 (38–40), GTDB r220 (41–44), and Greengenes2 2024.9 (39, 45) and by training new classifiers for GROND (18) and MIrROR (17). To account for unequal read depth between methods, samples were rarefied to an even depth of 5,716 reads per sample. For phylogenetic analyses, the QIIME2 implementations of mafft and fasttree2 were used to generate a tree (46, 47). NMDS plots were visualized with microViz (48), and box plots were created with ggplot2 (49).

Using the core-metrics-phylogenetic function from the diversity plug-in in QIIME2, beta-diversity was calculated for all samples by weighted UniFrac (50), unweighted UniFrac (51), generalized UniFrac (52), Bray-Curtis dissimilarity (53), and Jaccard index, which differ in how they account for phylogenetic relationships and relative abundances of taxa (37). Differences between groups of samples were assessed using PERMANOVA calculated with the adonis package in QIIME2, which generated *R*^2^ and adjusted *P* values (54). Additionally, the core-metrics-phylogenetic function was used to calculate the alpha-diversity of all samples by Shannon diversity (55), Pielou’s evenness (56), and Faith’s phylogenetic diversity (57) at the ASV level, which calculated significance by the Kruskal-Wallis test. After collapsing taxa at the genus and species levels based on assignments with Greengenes2, the core-metrics function was additionally used to calculate results specifically for Shannon diversity and Pielou’s evenness. To compare differences in the relative abundances and taxonomic classifications between sequencing methods, descriptive statistics were calculated with ggpubr’s desc_statby tool, and significance was assessed with ggpubr’s compare_means tool (58). Ribotyping was done by using phyloseq to subset specific taxa and generate heatmaps of relative abundance. The code used is available at https://github.com/joerggraflab/Code-for-OSullivan-2026.

## RESULTS

### Use of a mock DNA community to evaluate StrainID accuracy

**Fig 2:**
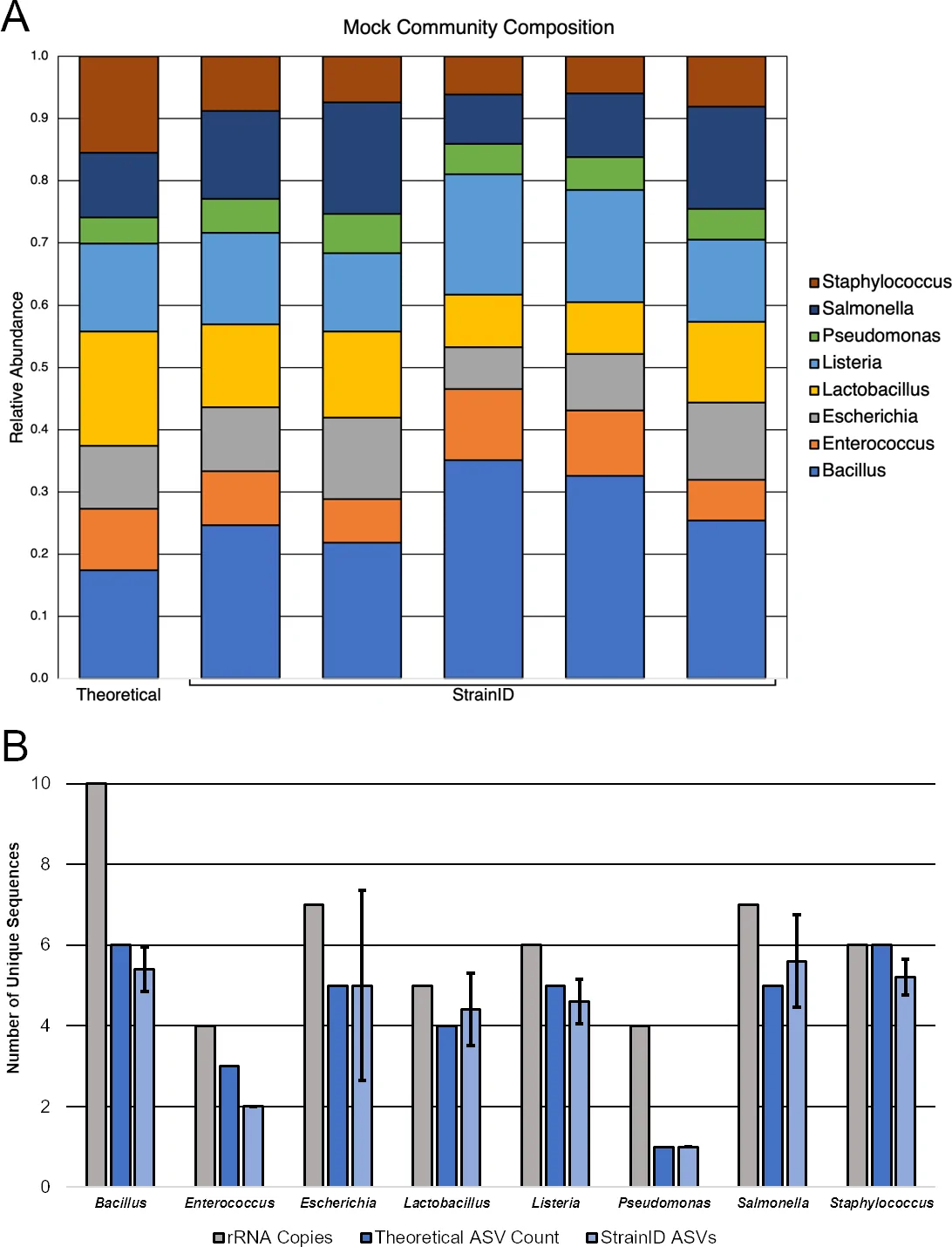
Analysis of a mock community with StrainID. (**A**) The composition of a defined DNA community against the predicted composition, shown with genus-level classifications. The mock community was amplified five separate times and sequenced across four different runs. (**B**) Mean number of ASVs for each taxa, compared to the expected number of ASVs and total rRNA operon copies in the reference genomes of all eight members of the mock community. Error bars represent one standard deviation.

To test for biases introduced by the StrainID primers, we evaluated a mock DNA community of eight taxa against the expected relative abundance. The mock community was amplified and sequenced five times across four sequencing runs. Across all sequencing runs, all eight genera were identified consistently at approximately the correct relative abundances reported by the manufacturer. The largest discrepancies were an overrepresentation of *Bacillus* (mean 0.28 ± 0.06 observed vs 0.174 expected) and an underrepresentation of *Staphylococcus* (mean 0.0727 ± 0.0121 observed vs 0.155 expected) (Fig. 2A). Additional analyses using a single replicate of the DNA mock community for the other amplicons also were performed (Fig. S1). The StrainID ASVs were bioinformatically truncated to correspond to full-length 16S and V1–V3 and received the prefix *in silico*. Both *in silico* amplicons derived from StrainID reads closely resembled the community structure with whole-length StrainID, indicating that differences relative to the theoretical values are driven by primer bias. Notably, the V1–V3 amplicon data were in poor agreement with the theoretical composition, with *Lactobacillus* and *Staphylococcus* each representing approximately 1% of the observed community (Fig. S1). Poor amplification of *Lactobacillus* with V1–V3 is consistent with known results (59).

Each member of the mock community had multiple copies of the rRNA operon in its genome, with as many as 10 copies in *Bacillus subtilis*. Sequences including more of the operon showed more of the sequence diversity between copies. As such, longer amplicons would be expected to result in additional ASVs for each taxon. Using the published genomes for the strains in the mock community, we identified the number of rRNA copies and how many unique sequences were present over the length of the StrainID amplicon, establishing a theoretical ASV count to compare against (Fig. 2B). Comparisons to shorter amplicons demonstrated that as sequence length increased, the number of ASVs predicted and observed increased (Fig. S2). The number of ASVs observed with StrainID closely matched the predicted number, which was higher than the ASVs for V1–V3 (Fig. 2B; Fig. S2).

### Phylum-level classification of bacterial communities

**Fig 3:**
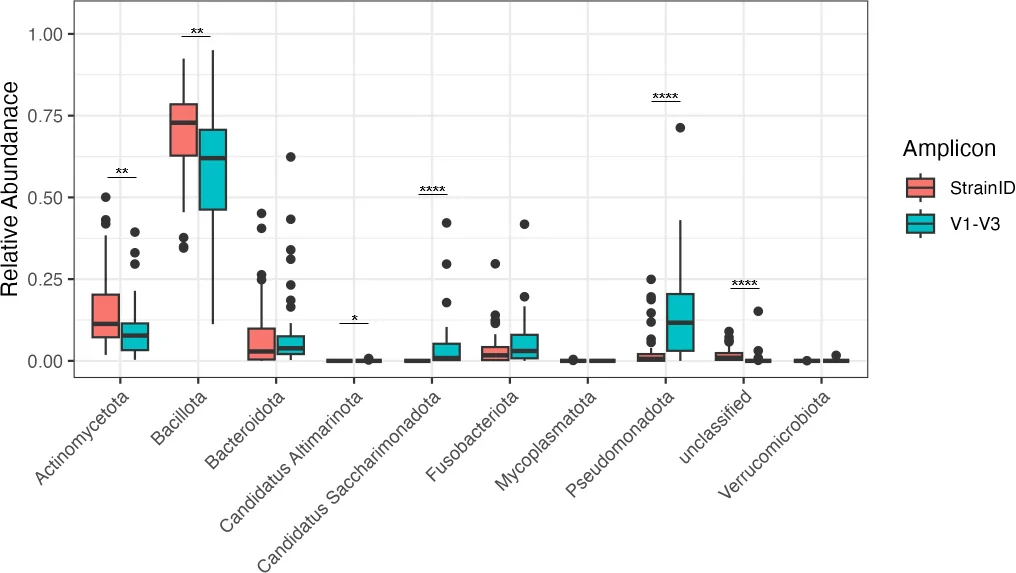
Phylum-level abundances of samples. Box and whisker plots of the relative abundances of the phyla observed in saliva samples. Horizontal bars represent median abundances (*P < 0.05, **P < 0.005, ****P < 0.00005).

After confirming the accuracy of StrainID with the mock community, we sought to compare its performance to SRA with 46 salivary samples at higher taxonomic levels. The overall compositions of the microbial communities were compared between the different amplicons. At the phylum level with classification using the Athena database, differences were observed between V1–V3 and StrainID in the saliva samples. This was not unexpected based on the observed primer bias in the mock community. Although Bacillota were the most dominant phylum with both methods, their overall abundance was higher with StrainID compared to V1–V3 (median abundance of 72.8% vs 62.0%, respectively; *P* < 0.05) (Fig. 3). The relative abundances of Actinomycetota, Pseudomonadota, and Saccharimonadota also differed significantly between V1–V3 and StrainID (*P* < 0.05). Notably, Saccharimonadota was not detected with StrainID. Analysis of the genome for an oral isolate of Saccharimonadota, *Nanosynbacter lyticus* TM7x (CP040011.1), revealed that the StrainID reverse primer had several mismatches and may fail to bind (60). Furthermore, in these taxa, the operon is non-contiguous due to the presence of *pyrD* and a gene encoding a hypothetical protein between the 16S and 23S rRNA genes. The resulting 7,069 bp amplicon is likely too long for the amplification conditions used and/or would be discarded during standard bioinformatic quality control steps. While the general patterns were consistent between the two amplicons, potentially biologically relevant differences were observed with StrainID.

### Taxonomic classification improves with StrainID

**Fig 4:**
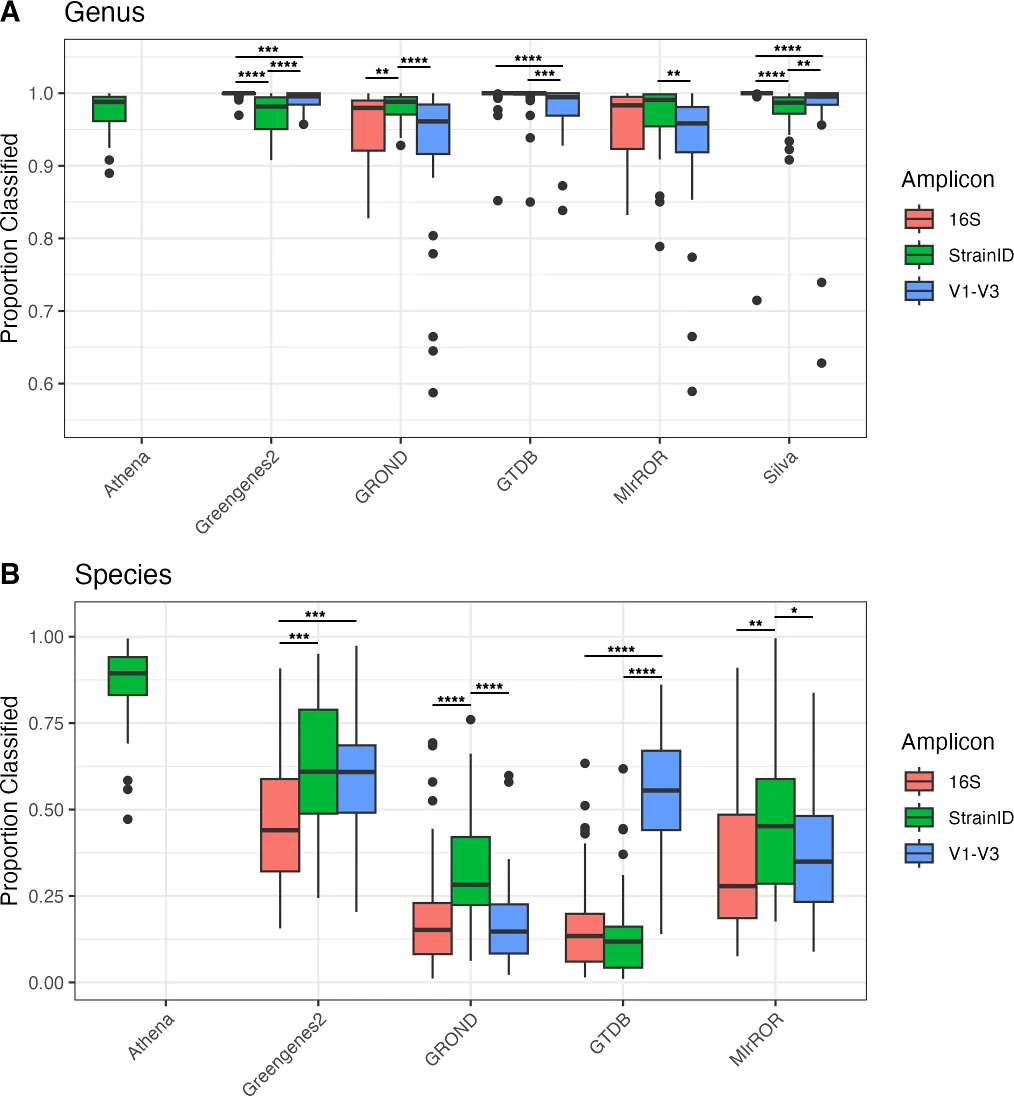
Read classification by amplicon with StrainID, V1-V3, and *in silico* 16S. Box and whisker plots for the proportion of reads classified at the genus (**A**) and species (**B**) levels with a variety of 16S and 16S-ITS-23S rRNA databases. Horizontal bars represent the median. Differences between amplicons were calculated by Wilcoxon test (*P < 0.05, **P < 0.005, ***P < 0.0005, ****P < 0.00005).

Performance of read classification at different taxonomic levels was evaluated with a mix of databases. Standard 16S rRNA databases such as Silva, Greengenes2, and GTDB were assessed using pretrained QIIME2 classifiers. For 16S-ITS-23S databases, GROND and MIrROR classifiers were trained in QIIME2, and Athena classification was done using the SBAnalyzer software packaged with StrainID. Generally, all amplicon and databases resulted in a high degree of successful genus-level assignment (Fig. 4A). The worst performers were V1–V3 with the MIrROR and GROND databases, at 93.4% ± 8.2% and 92.8% ± 9.3%, respectively. All other combinations resulted in a genus-level classification for 95.9% of reads or better. StrainID performance ranged from 98.2% ± 1.7% with GTDB to 96.8% ± 4.6% with MIrROR.

At the species level, performance depended strongly on whether the database was 16S only, or if it was combined 16S-ITS-23S (Fig. 4B). The best performance was obtained with StrainID and the Athena database with an average 87.0% ± 11.5% of the reads identified to the species level. Because SBAnalyzer uses fixed cutoff values for the species and strain identification based on the 16S-ITS-23 amplicon, using this classifier for shorter reads produces spurious results. The next best results were obtained with Greengenes2, and StrainID slightly outperformed V1–V3 (61.8% ± 21.6% and 59.8% ± 17.5%, respectively), though the difference was not statistically significant, and both amplicons outperformed the *in silico* 16S (*P* ≤ 0.0002). Interestingly, with the GTDB classifier, V1–V3 drastically outperformed both StrainID and the *in silico* 16S amplicons, with over a fourfold greater rate of species-level assignment. However, as both Greengenes2 and GTDB do not contain the ITS region and 23S rRNA, the strain ID amplicon cannot be aligned fully. The strength of StrainID was most apparent with the 16S-ITS-23S databases. With both GROND and MIrROR, StrainID resulted in significantly more reads classified than V1–V3 or *in silico* 16S (*P* ≤ 0.0003). Because Silva is not curated at the species level, the default for QIIME2 is to exclude species classifications for Silva to avoid incorrect or misleading assignments. Overall, these results demonstrate that StrainID excels at identifying reads to the species level when using Athena and SBAnalyzer.

The Athena database and the SBAnalyzer classifier are designed for StrainID and use fixed cutoffs for taxonomic assignment. Specifically, a 97% identity is used as a threshold for a strain-level classification and 95% identity for a species-level classification. These numbers are lower than traditionally used with the 16S rRNA gene only, due to the increased length and presence of the highly variable ITS region. As such, Athena is incompatible with shorter reads, as it will overclassify and lead to false positives. For StrainID, Athena resulted in the highest proportion of reads classified at the genus, species, and strain levels (Fig. S3).

### StrainID increases statistical power of diversity metrics

**Fig 5:**
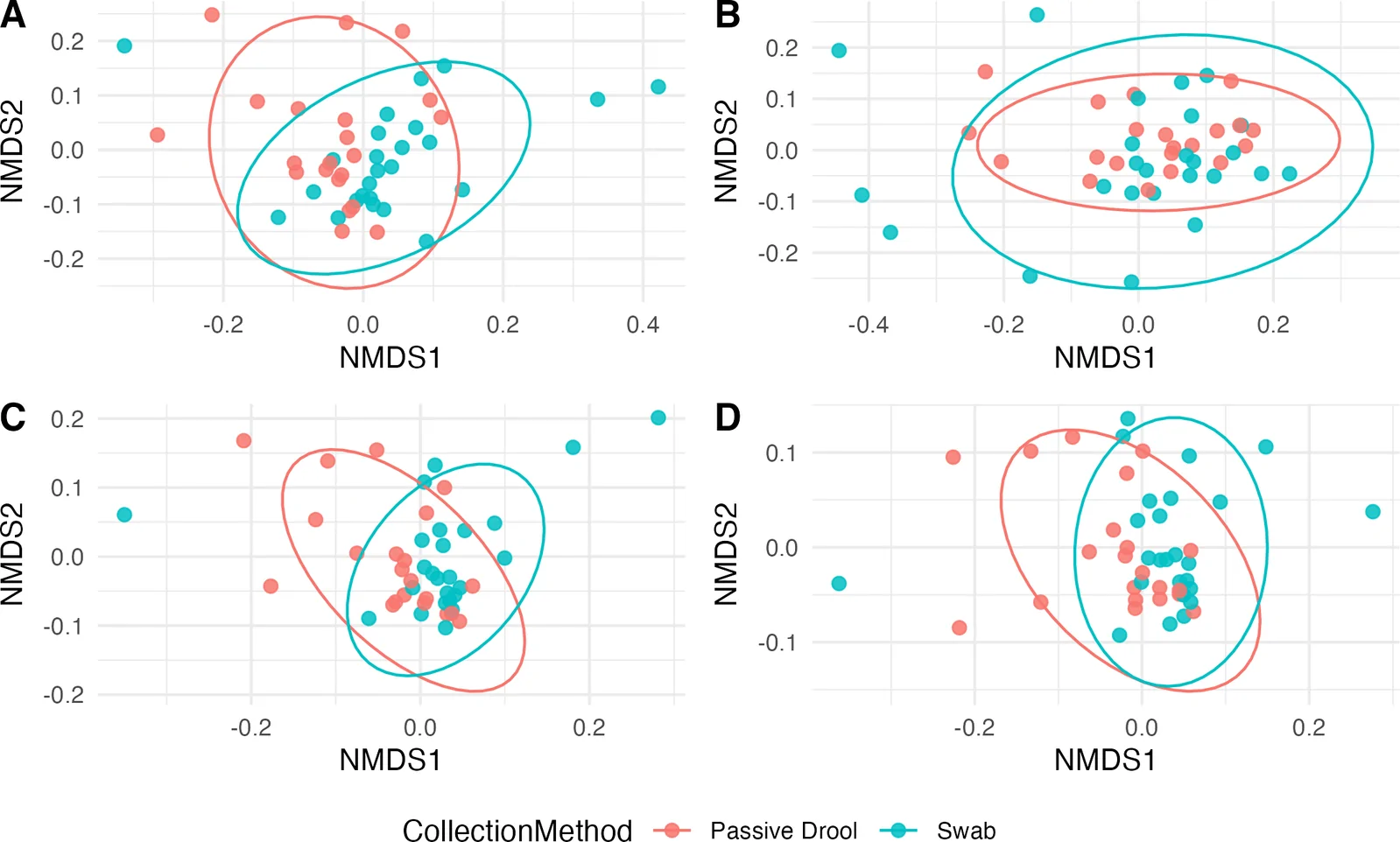
NMDS plots of weighted UniFrac for saliva samples. NMDS plots of StrainID (**A**), V1–V3 (**B**), *in silico* 16S (**C**), and *in silico* V1–V3 (**D**). Samples were grouped by the saliva collection method, and confidence intervals are denoted by ellipses.

To assess the practical application of the different approaches, we next sought to test the ability to differentiate between two different saliva collection methods, passive drool and swab. Children 6 years of age or older were sampled using a passive drool method, while children younger than 6 had their saliva collected using a swab. We used five different beta-diversity metrics: (i) Bray-Curtis dissimilarity, which accounts for relative abundance but not phylogenetic relationships; (ii) Jaccard, which ignores phylogeny and is based on presence and absence; (iii) weighted UniFrac, which accounts for both phylogeny and relative abundance; (iv) unweighted UniFrac, which is a phylogenetic presence-absence metric; and (v) generalized UniFrac, which is a middle ground between weighted and unweighted UniFrac. We assessed if there were differences in the salivary microbiome, depending on the collection method, using PERMANOVA calculated with the adonis tool in QIIME2. The largest amount of variation explained by the collection method was with the StrainID amplicon and weighted UniFrac (*R*^2^ = 0.064, *P* = 0.002) (Fig. 5A). With the V1–V3 primer set, this comparison was both not significant and explained less variation (*R*^2^ = 0.021, *P* = 0.471) (Fig. 5B). The *in silico* approaches performed similarly to StrainID, though the differences between collection methods were slightly less significant (16S *R*^2^ = 0.058, *P* = 0.018; V1–V3 *R*^2^ = 0.062, *P* = 0.011) (Fig. 5C and D).

A similar pattern was observed with generalized UniFrac. Both StrainID and the *in silico* amplicons resulted in significant differences (*P* ≤ 0.002) and *R*^2^ values between 0.061 and 0.065, whereas V1–V3 explained less variation between collection (*R*^2^ = 0.035), and the difference was not significant (*P* = 0.069) (Fig. S4). All other comparisons were significant (*P* < 0.05) and explained between 2.5% and 5.5% of variation between samples (Table 2).

**TABLE 2:** Adonis PERMANOVA of saliva collection method

	Weighted UniFrac		Unweighted UniFrac		Generalized UniFrac		Bray-Curtis dissimilarity		Jaccard index	
	*P*	*R* ^2^	*P*	*R* ^2^	*P*	*R* ^2^	*P*	*R* ^2^	*P*	*R* ^2^
StrainID	0.002	0.064241	0.003	0.055193	0.001	0.063462	0.003	0.037959	0.001	0.041695
V1–V3	0.471	0.021016	0.009	0.044536	0.069	0.034571	0.017	0.029916	0.039	0.025603
*In silico* 16S	0.018	0.058236	0.003	0.051639	0.002	0.060555	0.002	0.04091	0.001	0.041632
*In silico* V1–V3	0.011	0.062195	0.012	0.042507	0.001	0.064801	0.003	0.033964	0.004	0.027382

Additionally, we tested for differences in alpha-diversity between collection methods by Shannon diversity and Pielou’s evenness at the genus, species, and ASV levels, and Faith’s phylogenetic diversity specifically at the ASV level. Genus and species-level comparisons were done using taxonomic assignments with Greengenes2, as it performed the best across all samples. For both V1–V3 and *in silico* V1–V3, there was no difference in alpha-diversity by collection method. With longer amplicons, however, differences were observed. Samples differed significantly by collection method by Shannon diversity at the genus and species levels for both StrainID and *in silico* 16S (Table 3). Additionally, swab samples were more even than passive drool samples at the species level with *in silico* 16S (swab: 0.657 ± 0.097, passive drool: 0.582 ± 0.107, *P* = 0.016). At the ASV level, the only comparison that was statistically significant was StrainID with Faith’s phylogenetic diversity. Here, swab samples were significantly more diverse than passive drool samples (swab: 5.72 ± 1.66, passive drool: 4.73 ± 0.80; *P* = 0.041).

**TABLE 3:** Alpha-diversity statistics^*a*^

		Shannon diversity			Pielou’s evenness			Faith’s phylogenetic diversity		
		Swab (±SD)	Passive drool (±SD)	*P* value	Swab (±SD)	Passive drool (±SD)	*P* value	Swab (±SD)	Passive drool (±SD)	*P* value
ASV level	StrainID	5.426473 (1.246911)	4.86774 (0.877785)	0.083427	0.784273 (0.110649)	0.749918 (0.097811)	0.168112	5.719993 (1.657697)	4.733278 (0.798455)	**0.041363**
	V1–V3	4.690350 (0.907521)	5.059393 (0.728940)	0.120014	0.791114 (0.105390)	0.813024 (0.083926)	0.558953	9.259115 (2.050464)	10.12515 (1.662564)	0.125359
	*In silico* 16S	5.442726 (1.229949)	4.863637 (0.889697)	0.079569	0.786896 (0.107702)	0.74908 (0.09935)	0.175022	3.463383 (0.946079)	2.950741 (0.506655)	0.065563
	*In silico* V1–V3	4.985907 (0.84449)	4.635866 (0.577198)	0.10986	0.790143 (0.077486)	0.792697 (0.078829)	0.991202	5.854322 (1.443098)	5.617098 (1.182757)	0.558953
Species level	StrainID	2.727023 (0.661152)	2.276471 (0.548412)	**0.025198**	0.61595 (0.107422)	0.567105 (0.121033)	0.161409			
	V1–V3	3.428342 (0.679753)	3.596754 (0.536451)	0.395865	0.700306 (0.8843)	0.706524 (0.077207)	0.956033			
	*In silico* 16S	3.244562 (0.699139)	2.674137 (0.544767)	**0.006459**	0.657395 (0.097384)	0.581512 (0.106889)	**0.015745**			
	*In silico* V1–V3	3.576648 (0.654251)	3.275555 (0.497376)	0.095917	0.693982 (0.085866)	0.650968 (0.083793)	0.095917			
Genus level	StrainID	2.243976 (0.608167)	1.84378 (0.583415)	**0.025198**	0.549873 (0.112678)	0.494321 (0.132059)	0.083427			
	V1–V3	2.364706 (0.672649)	2.488003 (0.675135)	0.765923	0.570825 (0.126345)	0.571918 (0.125334)	0.851309			
	*In silico* 16S	2.181919 (0.607338)	1.818968 (0.561366)	**0.03333**	0.557637 (0.114012)	0.504326 (0.143161)	0.125359			
	*In silico* V1–V3	2.298734 (0.573608)	2.076447 (0.545009)	0.130888	0.557687 (0.103723)	0.513759 (0.118116)	0.100398			

^*a*^ Alpha-diversity of samples by collection method at the ASV level and after collapsing to the species and genus levels based on taxonomic assignment with Greengenes2. Differences between methods were calculated with a Kruskal-Wallis test. Statistically significant values are bolded.

### Ribotyping reveals shared patterns of ASVs across samples

Because most bacteria have multiple rRNA operon copies, they are expected to generate multiple ASVs with StrainID. A single ASV being present in multiple samples does not necessarily indicate the same strain is present, even with longer amplicons, because the other operon copies may have different sequences that are not shared between samples. Conversely, if the same sets of ASVs co-occur, it can be reasonably assumed that these samples share a specific ribotype. To evaluate the potential of ribotyping saliva samples, we generated heatmaps of ASVs belonging to specific taxa.

**Fig 6:**
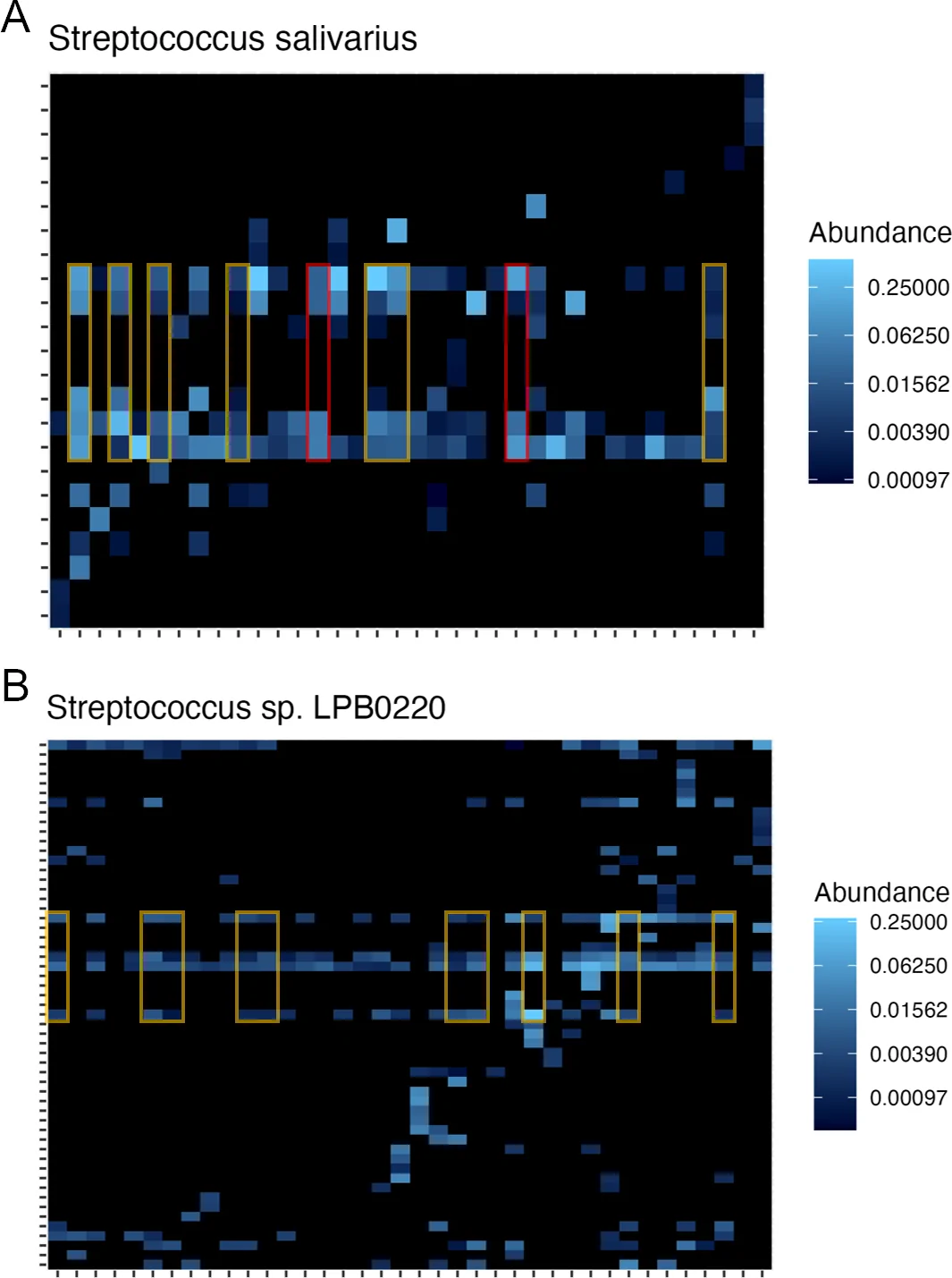
Ribotyping of *Streptococcus* species. Heatmaps showing the relative abundance for each ASV of *S. salivarius* (**A**) and *Streptococcus* sp. LPB0220 (**B**). Each row represents a different ASV, and each column represents an individual saliva sample. Red boxes highlight a set of ASVs comprising a ribotype. Yellow boxes are used to highlight shared sets of ASVs that may represent shared ribotypes in samples that appear to have multiple strains present.

It is not necessarily expected that samples from unrelated subjects share specific ribotypes, and previous work with fecal samples has shown that they often do not (16). The result is that in a complete data set, there are typically numerous ASVs present for abundant taxa, especially at broader classification levels with StrainID. Additionally, a single sample can have multiple different strains of the same species, all of which may belong to different ribotypes. As such, it is often difficult to identify ribotypes from dominant genera without subsetting individual species. We performed this analysis with *Streptococcus salivarius*, the overall most abundant species in the data set. The *rrn*DB estimates six total 16S rRNA copies in the *S. salivarius* genome (61). Because these copies are not necessarily unique sequences, six represents the theoretical maximum number of ASVs per ribotype. Several samples have more than six ASVs of *S. salivarius*, suggesting the presence of multiple ribotypes (Fig. 6A). Two samples each contain only the same four ASVs (Fig. 6A, denoted by red boxes). These same four ASVs co-occur in seven additional samples, which may be indicative of the same ribotype. However, because these occur alongside other ASVs, there are likely multiple ribotypes present, and it cannot be definitively determined that these four are all part of the same ribotype. We also examined *Streptococcus* sp. LPB0220, the third most abundant *Streptococcus* species and fifth most abundant species overall. With a predicted four rRNA copies by the *rrn*DB, we identified a set of four ASVs that co-occur in 13 samples, suggesting that the same ribotype is present (Fig. 6B). However, because most samples appear to contain multiple ribotypes of this species, it is again not possible to definitively conclude these are all the same ribotype.

**Fig 7:**
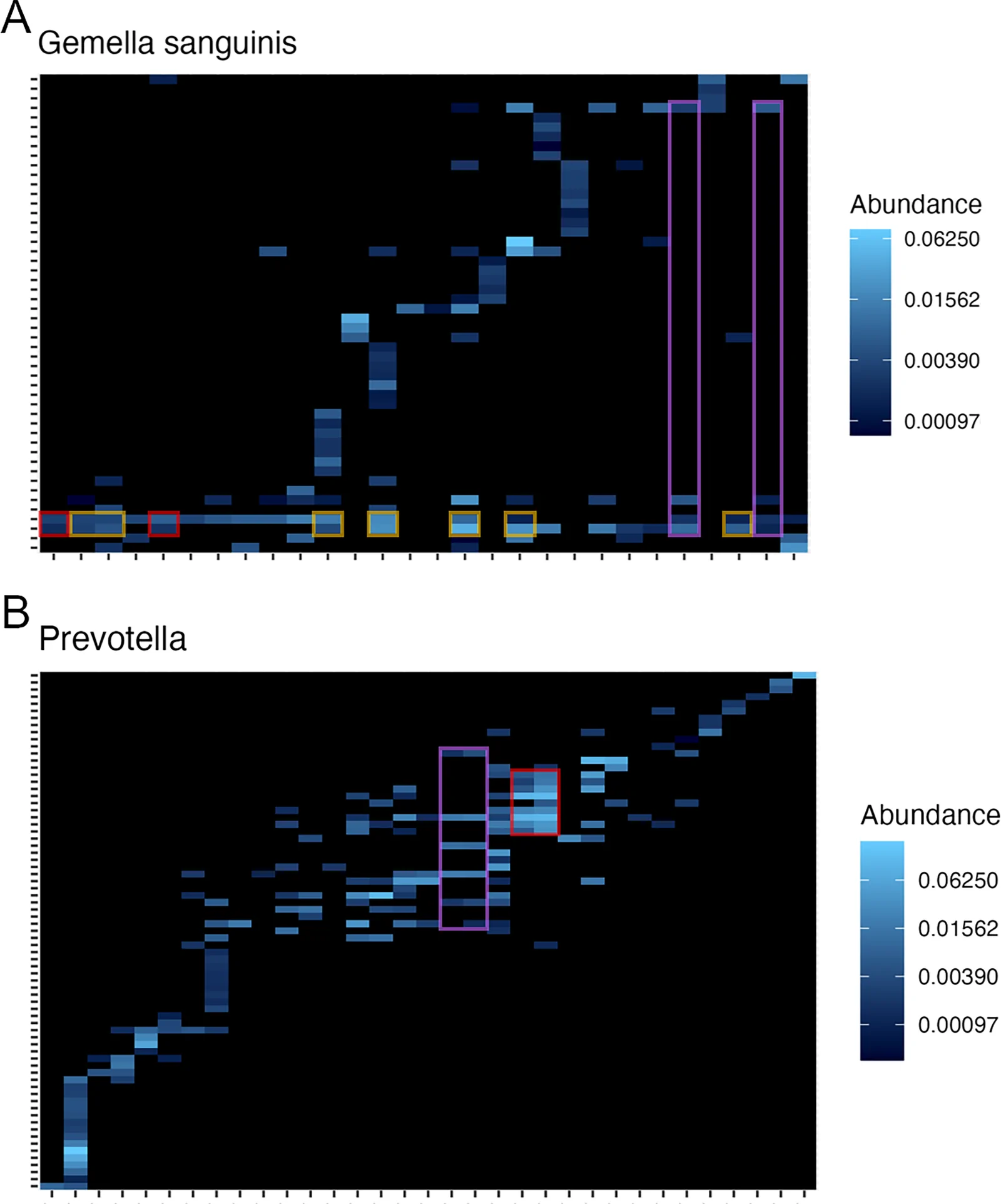
Ribotyping of abundant salivary taxa. Heatmaps showing the relative abundance for each ASV of *Gemella sanguinis* (**A**) and *Prevotella* (**B**). Each row represents a different ASV, and each column represents an individual saliva sample. Red and purple boxes highlight sets of ASVs comprising shared ribotypes. Yellow boxes are used to highlight sets of ASVs that may represent shared ribotypes in samples that appear to have multiple strains present.

For taxa outside of *Streptococcus*, we also examined *Gemella sanguinis* and *Prevotella. Gemella* was the fifth most abundant genus; *G. sanguinis* was the most abundant species in its genus and the 15th most abundant species overall. Here, we identified two distinct patterns of ASVs, each representing a distinct ribotype found in two samples (Fig. 7A). Up to seven additional samples potentially share one of these ribotypes, and the same pattern of ASVs was observed in addition to other ASVs (Fig. 7A). *Prevotella* was the third most abundant genus, but the majority of its ASVs were not identified to the species level with Athena. Because of this, we examined all ASVs that were part of the genus. For *Prevotella*, two distinct patterns were identified, each found in two samples (Fig. 7B). The first ribotype was composed of five ASVs. However, the second pattern was made up of eight ASVs shared in two samples. *Prevotella* is predicted to have between four and five rRNA copies; as such, this second cluster is likely composed of two different ribotypes that co-occur in these samples.

## DISCUSSION

Overall, these results demonstrate the usefulness of StrainID for use with human saliva samples in microbiome sequencing studies, not only by improving the ability to differentiate distinct sample types or cohorts but also by providing enhanced taxonomic insights. Additionally, they further establish StrainID as a robust methodology that compares favorably to traditional SRA approaches. In terms of taxonomic accuracy, StrainID exceeds the performance of V1–V3 for saliva samples. However, all primer sets introduce some bias (62, 63), and the ability to use one set of primers with excellent taxonomic resolution for all communities is another advantage of StrainID. The amount of primer bias seen with V1–V3 exceeded that observed with StrainID, suggesting that StrainID may be more accurate. Indeed, V1–V3 primers are known to poorly amplify taxa such as *Lactobacillus*, *Streptococcus*, and *Staphylococcus* relative to other primers, which could explain the lower overall abundance of Bacillota observed with V1–V3 (59, 64). One limitation of this study was that a generalized mock community was used. Although there was additional bias in this mock community with the V1–V3 primers, a community that more accurately reflects saliva could be used to provide direct insight into whether a particular primer set introduces more bias with actual samples. There is no loss in performance when comparing communities of samples with StrainID compared to SRA methods. Instead, StrainID was more effective than traditional approaches at distinguishing groups with phylogenetic alpha- and beta-diversity metrics. Of note, there were also significant differences in the performance of V1–V3 and *in silico* V1–V3, highlighting how differences in methodology can result in differences in data.

StrainID also provides the benefit of longer read length, which increased the accuracy of taxonomic assignment. Database selection is an important part of taxonomic classification and can significantly impact results (62, 63). Databases containing longer sequences are better able to provide matches with a high resolution but contain fewer sequences, which means certain taxa may be poorly represented, especially from understudied environments. We found that for a small proportion of ASVs, StrainID can fail to identify taxa even at the genus level more often than observed with *in silico* 16S approaches. These differences are likely driven by mismatches over the ITS region due to the failure of existing databases to capture the true diversity of sequences present in nature. While StrainID has the length to provide strain level classifications, it is dependent on a large enough database that includes strain-level information; current databases seem to be insufficient in this regard for saliva samples.

In addition, one can use the sequence information for the design of diagnostic primers or probes for fluorescent *in situ* hybridization. As the number of genomes in the public databases continuously increases, the accuracy of reference databases designed for long reads should increase as well and allow for even more sequences to be identified at the species level. When specifically comparing taxonomic assignment of saliva samples between V1–V3 and StrainID, we found that V1–V3 occasionally outperformed StrainID. However, StrainID generally was the most optimal amplicon for species-level assignments, especially with Athena and the SBAnalyzer classifier.

StrainID is not without shortcomings, which must be considered when selecting primers. For one, the amplicon design will fail to identify any bacteria with non-continuous rRNA operons, such as in *Helicobacter*, in which the 16S rRNA gene is not linked to the 23S and 5S rRNA genes (65). A similar case was seen in our data set with the phylum Saccharimonadota that was only detected with SRA due to incompatibility with StrainID. Saccharimonadota is a potentially significant phylum for saliva, and past studies have correlated this taxon with disease states (66). However, all known Saccharimonadota are episymbionts of other bacteria (60). As such, it is possible that any signal associated with specific species of Saccharimonadota could be identified by their symbionts, which likely would be compatible with StrainID. That StrainID will result in the loss of some taxa is a significant limitation of the approach and must be balanced against the benefits of improved resolution.

Additionally, the long sequence length of StrainID can lead to a low percentage of non-unique sequences. Because DADA2 relies on repeat observations, this can lead to excess reads being lost unless “pooling” the sequences from multiple samples together is done, which greatly increases the required computational time and power. Additionally, at the time of this publication, the cost per sample with StrainID is typically higher than with SRA methods due to limits in multiplexing. SRA barcodes support up to 384-plex sequencing runs. To date, StrainID is limited to a maximum of 112 unique barcodes. Further development and verification of additional barcodes for StrainID could help minimize this weakness. Adapting the primers to be compatible with PacBio’s Kinnex approach, which concatenates multiple amplicons together in a single molecule, could further assist in increasing the number of samples per run, in addition to increasing the average read depth, bringing the costs of StrainID more in line with SRA sequencing.

StrainID presents several important strengths. Our results suggest that StrainID improves taxonomic resolution and assignment accuracy when studying the microbiome. This is especially important for detecting differences in microbiome composition between different cohorts in clinical studies. Additionally, rRNA fingerprinting with StrainID allows identification of ribotypes (16). This is especially powerful in studies where tracking an individual ribotype would be useful, such as tracking transmission, outbreak, or temporal studies where continued presence of a ribotype would suggest its stability as a community member (67). This is possible only when there are multiple unique rRNA operon sequences in a genome, generating multiple unique ASVs from a single organism. With traditional short-read amplicons, multiple unique ASVs typically are not generated, but they are with StrainID. As such, the number of ASVs cannot be interpreted as the number of unique taxa present for StrainID but can be used to improve resolution. Herein, we demonstrated the ability to identify potential ribotypes of dominant taxa in saliva samples.

In conclusion, StrainID is accurate, matches or exceeds the performance of SRA, and improves taxonomic resolution, as has been demonstrated with longer amplicons for other sample types in the past (68). Compared to whole 16S rRNA sequencing, StrainID provides the added benefit of ribotyping. Given the advantages of this methodology, long-read sequencing approaches should be given consideration when designing a study where resolution is especially important. In recent years, there has been a greater shift towards the use of metagenomics in microbiome research due to the completeness of the data it provides compared to SRA. However, whole 16S rRNA sequencing is also becoming more common, especially as Oxford Nanopore’s accuracy continues to increase and PacBio’s cost per sample decreases. StrainID can be used in lieu of whole-length 16S rRNA sequencing to increase resolution for a similar cost and represents a more powerful approach for microbiome studies.

## Data Availability

Raw read data are available in the NCBI SRA database under project ID PRJNA1314305. Additional reads used can be found under project ID PRJNA910511 under BioSample IDs SAMN32127837–SAMN32127842 and SAMN32127844–SAMN32127851.

## SUPPLEMENTAL MATERIAL

Supplemental material spectrum.02776-25-s0001.docx Table S1; Fig. S1 to S4.

ASM does not own the copyrights to Supplemental Material that may be linked to, or accessed through, an article. The authors have granted ASM a non-exclusive, world-wide license to publish the Supplemental Material files. Please contact the corresponding author directly for reuse.
