# Supplementary material for: Comparison of a long-read amplicon sequencing approach to short-read amplicons for microbiome analysis
Source: Microbiol Spectr. 2026 May 19;14(7):e02776-25. doi: 10.1128/spectrum.02776-25 (PMC13349200; doi:10.1128/spectrum.02776-25)
Supplement: Supplemental material — Table S1; Fig. S1 to S4. [file spectrum.02776-25-s0001.docx]

**Supplemental Table 1:** Summary statistics of read counts for StrainID and V1-V3 after ASV calling.

|  | StrainID | V1-V3 |
| --- | --- | --- |
| Maximum | 42,151 | 92,976 |
| Minimum | 5,909 | 22,141 |
| Mean | 17,123 | 44,021 |
| Q1 | 9,070.25 | 30,655.75 |
| Median | 12,947.5 | 38,847.5 |
| Q3 | 25,123 | 51,666 |


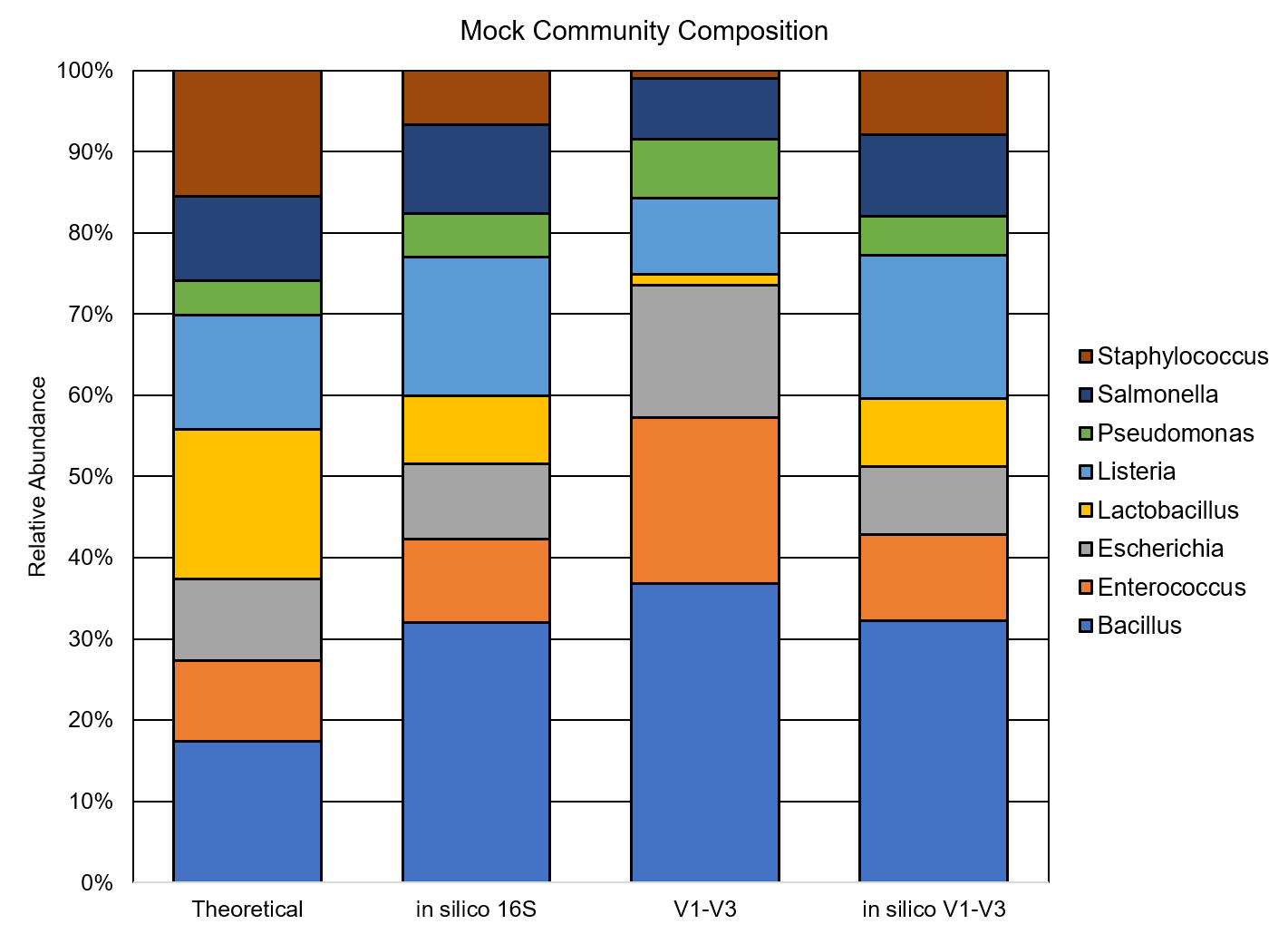


**Supplemental Figure 1:** The composition of a defined DNA community against the predicted composition, shown with genus-level classifications for each amplicon type tested, excluding StrainID. The *in silico* amplicons were derived from StrainID reads that were trimmed to the appropriate length prior to ASV calling.


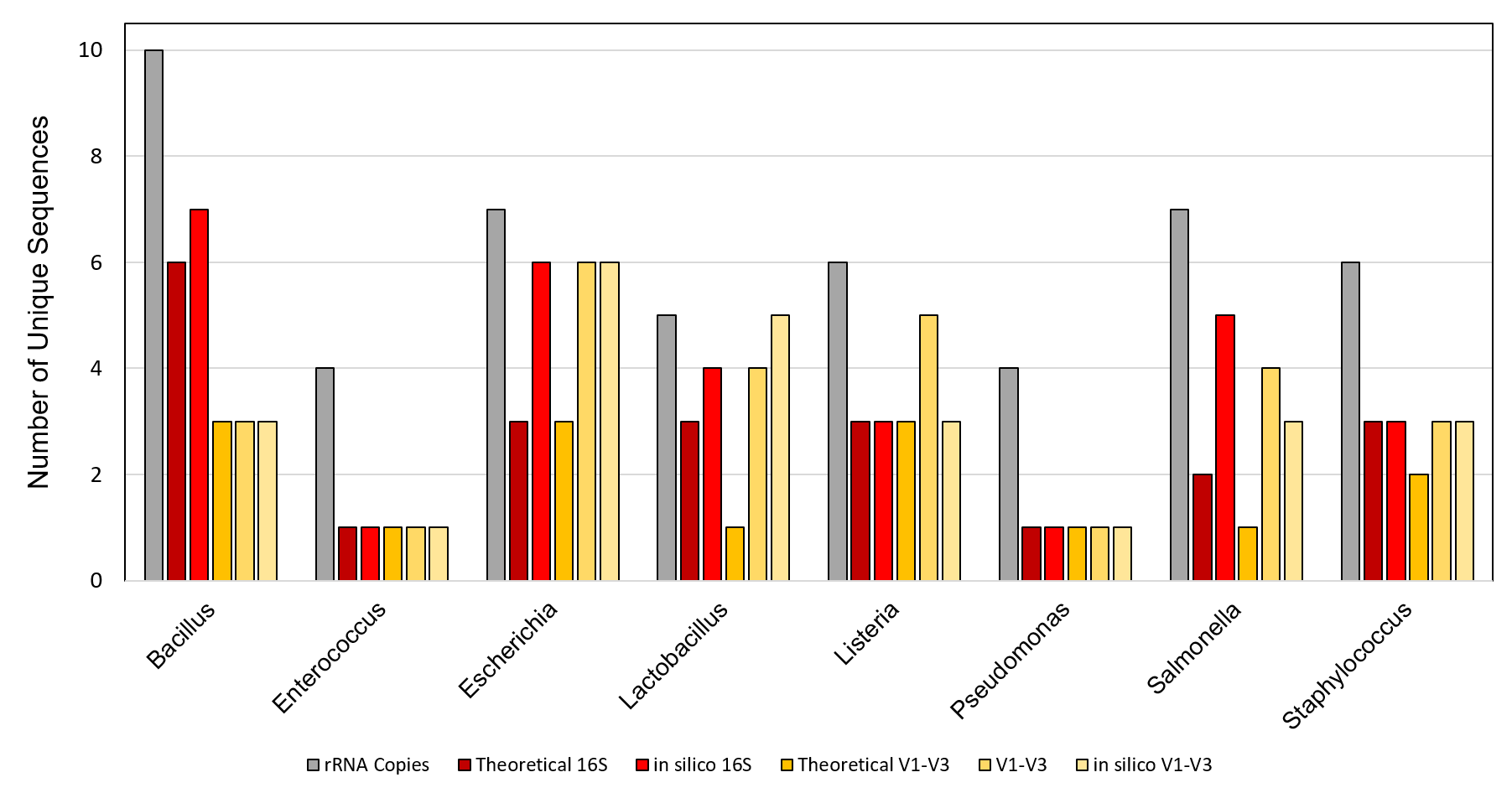


**Supplemental Figure 2:** The number of ASVs for each taxa, compared to the expected number of ASVs and total rRNA operon copies in the reference genomes of all 8 members of the mock community for all amplicon types tested.


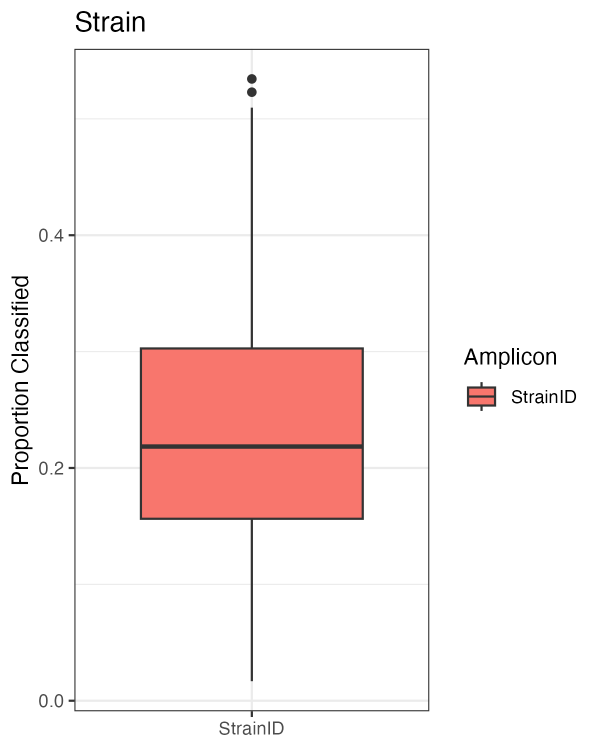


**Supplemental Figure 3:** Box and whisker plot for the proportion of reads classified at the strain level using the Athena database for StrainID. Horizontal bar represents the median abundances.


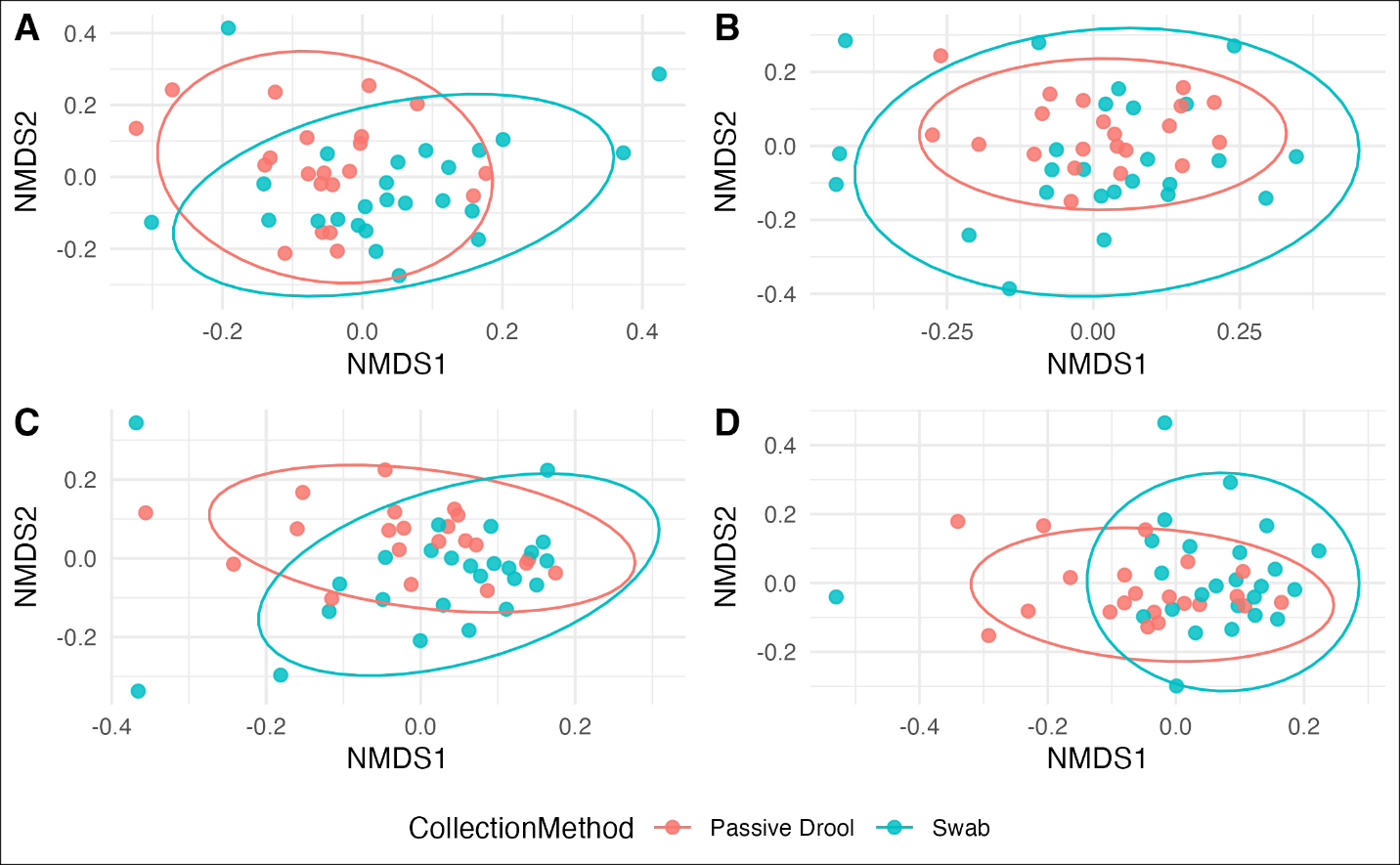


**Supplemental Figure 4:** NMDS plots of Generalized UniFrac for saliva samples. NMDS plots of StrainID (A), V1-V3 (B), *in silico* 16S (C), and *in silico* V1-V3 (D). Samples were grouped by the saliva collection method, and confidence intervals are denoted by ellipses.
